# Prevalence of anisometropia and associated factors in Shandong school-aged children

**DOI:** 10.3389/fpubh.2022.1072574

**Published:** 2022-12-22

**Authors:** Zihang Xu, Ziyun Wu, Ying Wen, Meihua Ding, Wei Sun, Yirong Wang, Zhen Shao, Yi Liu, Mingkun Yu, Guoyong Liu, Yuanyuan Hu, Hongsheng Bi

**Affiliations:** ^1^Ophthalmology and Optometry Medical School, Shandong University of Traditional Chinese Medicine, Jinan, China; ^2^Shandong Academy of Eye Disease Prevention and Therapy, Shandong Provincial Key Laboratory of Integrated Traditional Chinese and Western Medicine for Prevention and Therapy of Ocular Diseases, Shandong Provincial Clinical Research Center of Ophthalmology and Children Visual Impairment Prevention and Control, Shandong Engineering Technology Research Center of Visual Intelligence, Shandong Academy of Health and Myopia Prevention and Control of Children and Adolescents, Jinan, China; ^3^Affiliated Eye Hospital of Shandong University of Traditional Chinese Medicine, Jinan, China

**Keywords:** anisometropia, school-based study, associated factors, myopia, children

## Abstract

**Objective:**

To investigate anisometropia's prevalence and associated factors in school-aged children.

**Methods:**

A cross-sectional school-based study was conducted in Shandong Province, China, including children aged 4 to 17 from 9 schools. Anisometropia was defined as the differences between the two eyes in spherical equivalent (SE) or cylinder degree of 1.00 diopter (D) or more [SE or cylindrical (CYL) difference ≥ 1.00 D] after cycloplegic autorefraction. The Generalized Linear Model (GLM) was used to analyze the effects of ocular parameters [the differences between eyes in axial length (AL), habitual visual acuity (HVA), and corneal astigmatism (CA)] and lifestyle parameters (time spent indoor near work and outdoor activities) on anisometropia.

**Results:**

Total 4,198 (93.4%) of the 4,494 children were included in the statistical analysis. The mean difference in inter-eye SE was 0.42 ± 0.61 D. The prevalence of anisometropia was 13.2% (95%CI: 12.1 to 14.2%) (SE anisometropia's prevalence:10.3%; CYL anisometropia's prevalence: 4.1%), increased with older age (OR = 1.10, *P* = 0.002), the worse myopic eye (myopia vs. premyopia, OR = 1.87, *P* = 0.002), the worse hyperopic eye (hyperopia vs. premyopia, OR = 1.77, *P* = 0.013), larger difference in inter-eye AL (0.1–0.3 vs. ≤ 0.1, OR = 1.67, *P* = 0.008; >0.3 vs. ≤ 0.1, OR = 28.61, *P* < 0.001), HVA (>0.2 vs. ≤ 0.2, OR = 3.01, *P* < 0.001), CA (OR = 6.24, *P* < 0.001), the worse stereoacuity (>100 vs. ≤ 100, OR = 1.59, *P* = 0.001), longer indoor near work time per day on weekends (4–8 vs. <4, OR = 1.41, *P* = 0.038; ≥8 vs. <4, OR = 1.40, *P* = 0.131), and shorter outdoor activity time per day on weekdays (≥1 vs. <1, OR = 0.75, *P* = 0.046) in multivariable analysis. In the SE anisometropia group, the difference in inter-eye AL (>0.3 vs. ≤ 0.1, β: 0.556, 95%CI: 0.050 to 1.063), HVA (>0.2 vs. ≤ 0.2, β: 0.511, 95%CI: 0.312 to 0.710), and CA (β: 0.488, 95%CI: 0.289 to 0.688), stereoacuity (>100 vs. ≤ 100, β: 0.299, 95%CI: 0.110 to 0.488) had a positive impact on the difference in inter-eye SE.

**Conclusions:**

Ocular parameters and lifestyle parameters are associated with the occurrence of anisometropia in children aged 4 to 17 years, including the difference in inter-eye AL, HVA, CA, stereoacuity, indoor near work time, and outdoor activity time. Preventing myopia and early treating anisometropic amblyopia may be effective ways to reduce the prevalence of anisometropia.

## Introduction

Anisometropia, a difference in the refractive error between two eyes, is a common cause of poor binocular vision and amblyopia, which is widely popular among all ages. A patient with anisometropia may also have symptoms of diplopia, aniseikonia, and decreased stereoacuity, which might severely affect binocular dysfunction. Anisometropia may cause visual fatigue, difficulty in binocular image fusion, monocular macular central fovea depression, or decreased stereoacuity, which should be paid more attention (1–3).

Both eyes of the individual have the same genetic background and are influenced by the same environment, but they may develop different refractive status due to asymmetric growth. Studies have confirmed that both ocular parameters and lifestyle parameters are closely related to the occurrence of anisometropia (4). However, the etiology and pathogenesis of anisometropia have not been well-studied. The key to reducing its harm remains early detection and control of its further progression (5). Thus in the current study, we aimed to investigate the prevalence of anisometropia in children and assess the impact of potential factors based on a cross-sectional school-based study.

## Materials and methods

### Study participants

In September 2020, we conducted a cross-sectional study based on a multistage stratified cluster random sampling in Huantai City, Shandong Province, China. Total of 4,494 children from 9 schools (2 kindergartens, 4 primary schools, 2 middle schools, and 1 high school) participated in the survey. Children with eye diseases, such as nystagmus, fundus diseases, and any history of eye surgery, were excluded.

### Study examinations

All children underwent examinations at school, including an interview with a standardized questionnaire similar to the one used in the RESC (Refractive Error Study in Children) studies. The questionnaire included basic demographic data such as age, gender, parental refractive status, and lifestyle parameters such as indoor near work time and outdoor activity time.

Firstly, two ophthalmologists performed slit-lamp examinations and fundus examinations for all children before the study, to exclude children with eye diseases that affected this study. All other examinations were performed by professional optometrists, including habitual visual acuity (HVA) at a standard testing distance of 3 meters (#600722, Good-Lite Co., Elgin, IL, USA), non-contact tonometers (Topcon CT80; Topcon Corp., Tokyo, Japan), non-cycloplegic and cycloplegic autorefraction (Nidek ARK-1, CO., LTD, Japan) and Stereoacuity test (Titmus test, described below). Axial length (AL) was acquired using laser interferometry (IOL-Master 500, Carl Zeiss Meditec AG, Jena, Germany), measured 3 times, and the average of readings was calculated. The cycloplegic eye drops used in this study were 1% cyclopentolate hydrochloride Ophthalmic Solution (Alcon, Fort Worth, TX, USA), instilled once every 5 min for a total of 3 times. Then the subjects closed their eyes and rested for 30 min before observing pupil light response and diameter. If the pupillary light reflex disappeared or the pupil diameter was > 6 mm, an autorefractor was performed. If the pupil diameter is < 6 mm, another eye drop was put in. The cycloplegic refraction was acquired 10 min later.

Stereoacuity was assessed by using the Titmus test (Stereo Optical Co., Inc., Chicago, IL, USA). Titmus test ranged from 800 to 40 sec of arc. Wearing polarized glasses, the subjects viewed the fly at a distance of 40 cm and were asked to “pinch” the tip of a wing between the thumb and forefinger. If successful, they were asked to point to the circle that appeared ahead of the plane and seemed to come closer to them. If the subject made one mistake, the previous target was reassessed. If the subject made an accurate judgment on the previous one, we recorded the level of stereopsis. Other detailed examinations have been reported in previous studies (6).

### Definition

Spherical equivalent (SE) was defined as the sum of the spherical refractive error plus half of the cylindrical (CYL) refractive error (measured as minus values). SE and CYL anisometropia was defined as the difference in cycloplegic inter-eye SE or cylinder degree≥1.00 D, respectively. The differences in inter-eye SE and CYL were presented in the form of absolute values. According to the International Myopia Institute (IMI) (7), myopia was defined as SE ≤ −0.50 D after cycloplegia. Premyopia was defined as −0.50 D <SE ≤ 0.75 D after cycloplegia, and hyperopia was defined as SE > 0.75 D after cycloplegia. Myopic anisometropia was defined as an unequal amount of myopia in both eyes, and hyperopic anisometropia was defined as an unequal amount of hyperopia in both eyes. The worse eye refers to refractive error of the eye with the highest absolute refractive error. If both eyes had the same SE, the right eye was defined as the worse eye. According to the average of cycloplegic SE [(SE of the right eye + SE of the left eye)/2], the refractive status was divided into five groups, including moderate to high hyperopia (SE>2.00 D), low hyperopia (0.75 D<SE ≤ 2.00 D), premyopia, low myopia (−3.00 D <SE ≤ −0.50 D), and moderate to high myopia (SE ≤ −3.00 D).

### Statistical analysis

All data were analyzed using the SPSS 25.0 software (Inc Chicago, IL). Measurement data were given as mean ± standard deviation (M ± SD) and median. Enumeration data were expressed as cases/percentage [N (%)] and 95% confidence interval (CI). Independent-Samples *t*-test was used to compare the differences between groups. Categorical data were analyzed using the Chi-square test for variance and trend test (P_trend_). Continuous data were tested using polynomial linear correlation in one-way ANOVA for differences between multivariate groups and trend test (P_trend_). The influencing factor analysis uses Variance Inflation Factor (VIF) diagnostics to diagnose the multicollinearity of independent variables. The Generalized Linear Model (GLM) was used for multivariate analysis of all ocular parameters and lifestyle parameters with statistically significant differences (*P* < 0.050) in univariate analysis and VIF <4. The effect size of the covariates was based on GLM coefficients (β) with a 95% CI. Under a logistic model, odds ratio (OR) and 95% CI were computed to assess associated factors for the prevalence of anisometropia. All *P*-values were 2-sided and were considered statistically significant when the values were < 0.05.

## Results

Among 4,494 eligible children who were recruited in the cross-sectional study, 4,198 children [93.4%, 2,123 (50.6%) boys] were finally included in the statistical analysis, and 285 were excluded (283 with non-cycloplegia, 13 with incomplete refraction data). Among them, total of 432 [10.3%, 198 (48.5%) boys] and 172 [4.1%, 98 (56.4%) boys] children were diagnosed with SE and CYL anisometropia, including 51 children (1.2%) were diagnosed with both SE and CYL anisometropia, and 3,645 children (86.8%) were included in the non-anisometropia group. Table 1 shows the distribution of basic demographic and ocular paramesters.

**Table 1:** Distribution of characteristics among all children.

**Associated factors**	**Total** **(*n* = 4198)**	**SE anisometropia group** **(*n* = 432)**	**CYL anisometropia group** **(*n* = 172)**	**Non-anisometropia group** **(*n* = 3766)**	***P*-value^a^**	***P*-value ^b^**
**Age (years)**	9.24 ± 3.15	11.98 ± 2.84	10.56 ± 3.52	8.93 ± 3.04	0.000	0.000
**Difference in inter-eye SE (D)**	0.42 ± 0.61	1.82 ± 1.01	0.99 ± 1.29	0.26 ± 0.22	0.000	0.000
**Difference in inter-eye CYL (D)**	0.27 ± 0.32	0.44 ± 0.47	1.31 ± 0.54	0.22 ± 0.20	0.000	0.000
**Difference in inter-eye CA (D)**	0.32 ± 0.33	0.20 ± 0.62	0.94 ± 0.64	0.06 ± 0.42	0.000	0.000
**Gender**						
Boy	2,123 (50.6%)	198 (45.8%)	97 (56.4%)	1,925 (51.1%)	0.038	0.119
Girl	2,075 (49.4%)	234 (54.2%)	75 (43.6%)	1,841 (48.9%)		
**Worse eye refractive status**						
Premyopia	1,369 (32.6%)	42 (9.7%)	39 (22.7%)	1,327 (35.2%)	0.000	0.000
Hyperopia	1,285 (30.6%)	57 (13.2%)	33 (19.2%)	1,228 (32.6%)		
Myopia	1,544 (36.8%)	333 (77.1%)	100 (58.1%)	1,211 (32.2%)		
**Difference in inter-eye AL (mm)**						
≤0.1	2,031 (48.4%)	15 (3.5%)	47 (27.3%)	1,972 (54.1%)	0.000	0.000
0.1–0.3	1,537 (36.6%)	49 (11.3%)	63 (36.6%)	1,433 (39.3%)		
>0.3	626 (14.9%)	367 (85.0%)	62 (36.0%)	237 (6.5%)		
**Difference in inter-eye HVA (LogMAR)**						
≤0.2	3,885 (92.5%)	281 (65.0%)	138 (80.2%)	3,604 (95.7%)	0.000	0.000
>0.2	313 (7.5%)	151 (35.0%)	34 (19.8%)	162 (4.3%)		
**Stereoacuity (arc-s)**						
≤100	2,904 (69.2%)	217 (50.2%)	97 (56.4%)	2,687 (71.4%)	0.000	0.000
>100	1,281 (30.5%)	212 (49.2%)	75 (43.6%)	1,069 (28.4%)		

a For comparison between SE anisometropia group vs. non-anisometropia group.

b For comparison between CYL anisometropia group vs. non-anisometropia group.

SE, Spherical equivalent; CYL, Cylinder; CA, Corneal astigmatism; AL, Axial length; HVA, Habitual visual acuity; D, Diopter; mm, Millimeter.

Due to the lack of cooperation in test, missing data occurred in 4 (0.1%) children for interocular difference in AL, and 13 (0.3%) children in stereoacuity.

**Figure 1:**
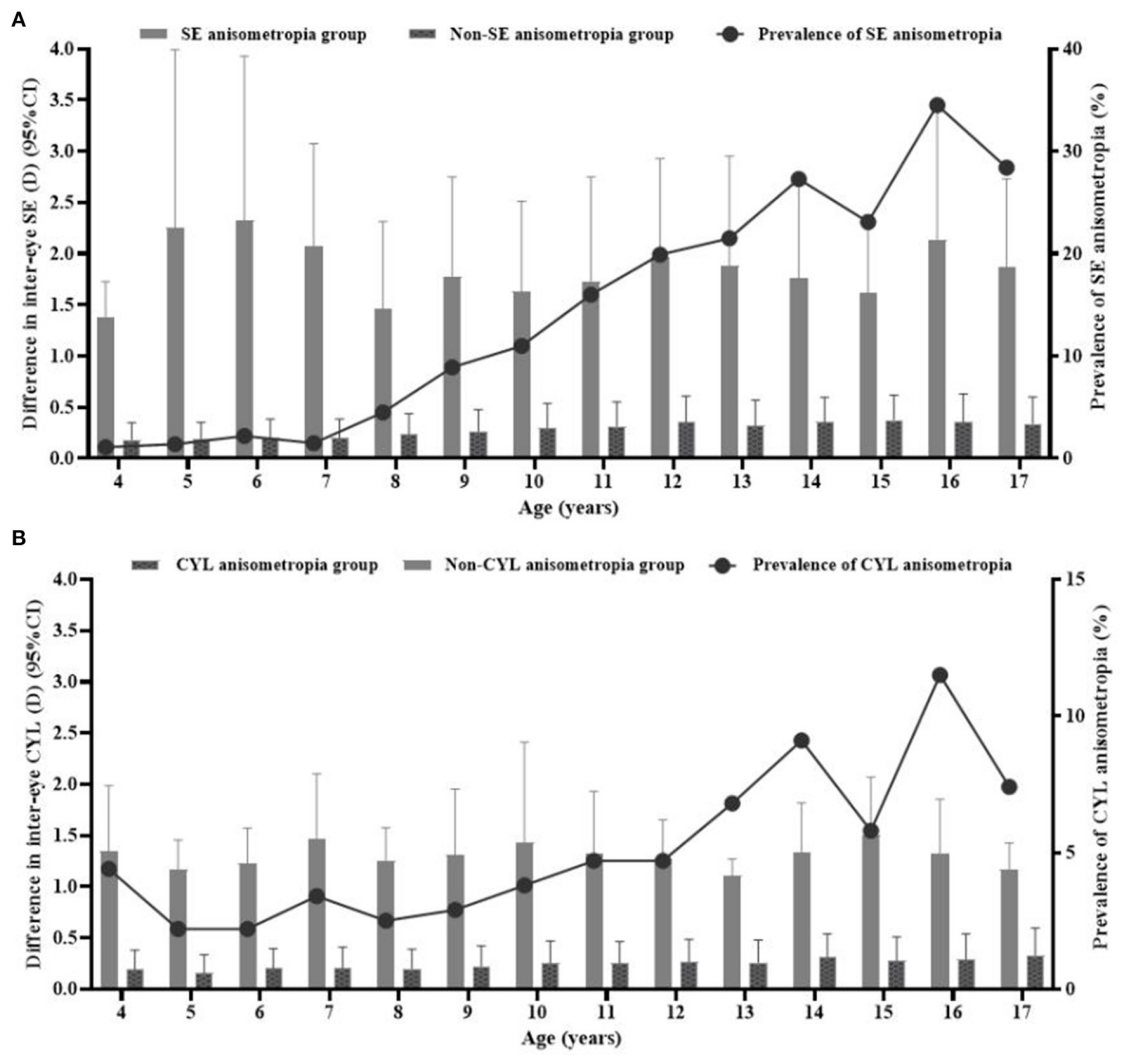
Distribution of the difference in inter-eye spherical equivalent (SE) and cylinder (CYL) and prevalence of SE **(A)** and CYL **(B)** anisometropia at different ages. The grey bars represented the difference in inter-eye SE and CYL in the anisometropia and Non-anisometropia groups (corresponding to the left y-axis). The black line represented the prevalence of SE and CYL anisometropia (corresponding to the right y-axis).

The mean difference in inter-eye SE and CYL was 0.42 ± 0.61D (median: 0.25D; range: 0–7.25D) and 0.27 ± 0.32D (median: 0.25D; range: 0–4.75D) among all children. With increasing age, the anisometropia's prevalence gradually increased, and the difference in inter-eye SE gradually widened (P_trend_ <0.001). No significantly different occurrence of anisometropia was found according to gender (χ^2^ = 1.336, P>0.050). The results are shown in Figure 1, Table 2. The multivariable logistic regression analysis showed the prevalence increased with older age (OR = 1.10, *P* = 0.002), the worse myopic eye (myopia vs. premyopia, OR = 1.87, *P* = 0.002), the worse hyperopic eye (hyperopia vs. premyopia, OR = 1.77, *P* = 0.013), larger difference in inter-eye AL (0.1–0.3 vs. ≤ 0.1, OR = 1.67, *P* = 0.008; >0.3 vs. ≤ 0.1, OR = 28.61, *P* < 0.001), HVA (>0.2 vs. ≤ 0.2, OR = 3.01, *P* < 0.001), CA (OR = 6.24, *P* < 0.001), the worse stereoacuity (>100 vs. ≤ 100, OR = 1.59, *P* = 0.001), longer indoor near work time per day on weekends (4–8 vs. <4, OR = 1.41, *P* = 0.038; ≥8 vs. <4, OR = 1.40, *P* = 0.131), and shorter outdoor activity time per day on weekdays (≥1 vs. <1, OR = 0.75, *P* = 0.046) (shown in Table 3).

**Table 2:** The prevalence of anisometropia at different age.

**Age**	**Number of children (%)**	**Number of SE anisometropia children (%)**	**Difference of inter-eye SE in SE anisometropia group (D)**	**Cycloplegic SE of the worse eye in SE anisometropia group (D)**	**Number of CYL anisometropia children (%)**	**Difference of inter-eye CYL in CYL anisometropia group (D)**
4	180 (4.3)	2 (0.5)	0.20 ± 0.21	1.31 ± 0.77	8 (4.4)	0.24 ± 0.33
5	139 (3.3)	2 (0.5)	0.22 ± 0.33	1.13 ± 0.74	3 (2.2)	0.18 ± 0.23
6	556 (13.2)	12 (2.8)	0.25 ± 0.42	1.03 ± 0.72	12 (2.2)	0.22 ± 0.25
7	596 (14.2)	9 (2.1)	0.23 ± 0.31	0.63 ± 1.07	20 (3.4)	0.25 ± 0.33
8	555 (13.2)	25 (5.8)	0.29 ± 0.37	0.17 ± 1.19	14 (2.5)	0.22 ± 0.26
9	450 (10.7)	40 (9.3)	0.40 ± 0.56	−0.02 ± 1.51	13 (2.9)	0.25 ± 0.29
10	372 (8.9)	41 (9.5)	0.45 ± 0.56	−0.73 ± 1.69	14 (3.8)	0.30 ± 0.36
11	319 (7.6)	51 (11.8)	0.54 ± 0.69	−1.06 ± 1.79	15 (4.7)	0.30 ± 0.33
12	301 (7.2)	60 (13.9)	0.68 ± 0.81	−1.61 ± 1.86	14 (4.7)	0.31 ± 0.31
13	205 (4.9)	44 (10.2)	0.66 ± 0.84	−2.24 ± 2.28	14 (6.8)	0.32 ± 0.30
14	253 (6.0)	69 (16.0)	0.74 ± 0.84	−2.90 ± 2.38	23 (9.1)	0.41 ± 0.39
15	104 (2.5)	24 (5.6)	0.66 ± 0.67	−3.45 ± 2.16	6 (5.8)	0.35 ± 0.39
16	87 (2.1)	30 (6.9)	0.97 ± 1.14	−3.72 ± 2.23	10 (11.5)	0.41 ± 0.44
17	81 (1.9)	23 (5.3)	0.78 ± 0.86	−3.92 ± 2.37	6 (7.4)	0.39 ± 0.34
Total	4,198 (100.0)	432 (100.0)	0.42 ± 0.61	−2.02 ± 2.53	172 (100.0)	0.27 ± 0.32
**χ^2^** *(*F)		363.457^*^	(38.491)	(281.513)	31.728^*^	(10.945)
*P*-value		0.000^*^	0.000	0.000	0.000^*^	0.000

* Results of Chi-square test for trend test (P_trend_).

SE, Spherical equivalent; CYL, Cylinder; D, Diopter.

**Table 3:** Logistic regression analysis assessing associated factors for the prevalence of anisometropia.

**Associated Factors**	**Number of children**	**Number of SE anisometropia children (%)**	**Univariate analysis**		**Multivariate analysis**	
			**OR (95%CI)**	***P*** **-value**	**OR (95%CI)**	***P*** **–value**
**Age (years)**	4,198	553 (13.2%)	1.29 (1.25 to 1.34)	0.000	1.10 (1.03 to 1.16)	0.002
**Refractive status of the worse eye**						
Premyopia	1,369	78 (5.7%)	Ref.		Ref.	
Hyperopia	1,285	83 (6.5%)	1.28 (0.90 to 1.82)	0.173	1.77 (1.13 to 2.77)	0.013
Myopia	1,544	392 (25.4%)	5.83 (4.38 to 7.76)	0.000	1.87 (1.26 to 2.76)	0.002
**Difference in inter-eye AL (mm)**						
≤0.1	2,031	59 (2.9%)	Ref.		Ref.	
0.1-0.3	1,537	104 (6.8%)	2.17 (1.52 to 3.10)	0.000	1.67 (1.14 to 2.45)	0.008
>0.3	626	390 (62.3%)	52.40 (37.71 to 72.81)	0.000	28.61 (19.85 to 41.25)	0.000
**Difference in inter-eye HVA (LogMAR)**						
≤0.2	3,885	387 (10.0%)	Ref.		Ref.	
>0.2	313	166 (53.0%)	10.41 (7.95 to 13.63)	0.000	3.01 (2.08 to 4.35)	0.000
**Difference in inter-eye CA (D)**	4,198	553 (13.2%)	6.28 (4.78 to 8.25)	0.000	6.24 (4.37 to 8.93)	0.000
**Stereoacuity (arc-s)**						
≤100	2,904	297 (10.2%)	Ref.		Ref.	
>100	1,281	256 (20.0%)	2.12 (1.73 to 2.58)	0.000	1.59 (1.21 to 2.10)	0.001
**Indoor near work time per day on weekends (hours)**						
<4	1,856	165 (8.9%)	Ref.		Ref.	
4-8	1,565	228 (14.6%)	1.92 (1.52 to 2.42)	0.000	1.41 (1.02 to 1.94)	0.038
≥8	777	160 (20.6%)	3.27 (2.52 to 4.25)	0.000	1.40 (0.91 to 2.17)	0.131
**Outdoor activity time per day on weekdays (hours)**						
<1	1,946	302 (15.5%)	Ref.		Ref.	
≥1	2,252	251 (11.1%)	2.09 (1.71 to 2.57)	0.000	0.75 (0.56 to 0.99)	0.046

Multivariable from logistic regression model were adjusted for gender, indoor near work time per day on weekdays (hours) and outdoor activity time per day on weekends (hours).

AL, Axial length; HVA, Habitual visual acuity; CA, Corneal astigmatism; OR, Odds ratio; CI, Confidence interval; D, Diopter; mm, Millimeter; Ref, Reference.

Due to the lack of cooperation in test, missing data occurred in 4 (0.1%) children for interocular difference in AL, and 13 (0.3%) children in stereoacuity.

**Figure 2:**
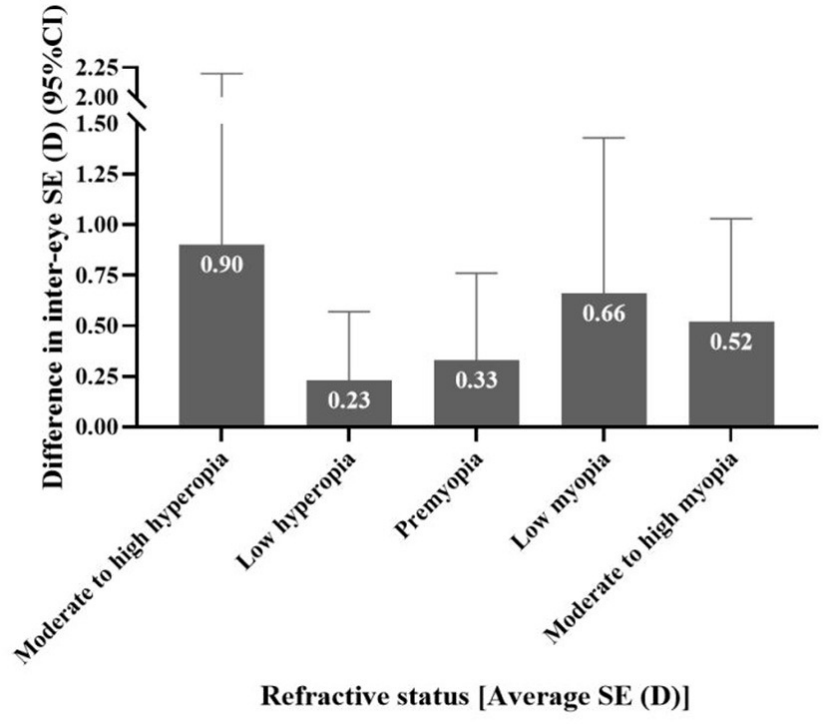
The distribution of the difference in inter-eye spherical equivalent (SE) in different refractive states among all children.

When comparing the difference in inter-eye SE in different refractive statuses among all children, it was found that the difference in inter-eye SE showed a U-shape curve. The difference was greater when in the stage of moderate to high hyperopia and moderate to high myopia. The difference decreased significantly from moderate to high hyperopia until low hyperopia and then showed a continuous upward trend at the premyopia and low myopia. Finally, at the stage of moderate to high myopia, the difference decreased slightly but stabilized (P_trend_ <0.001) (shown in Figure 2).

The multivariate GLM analysis showed ocular parameters and lifestyle parameters with a significant effect on the difference in inter-eye SE among all children (Table 4). The difference in inter-eye AL (0.1–0.3 vs. ≤ 0.1, β: 0.073, 95%CI: 0.004 to 0.106; >0.3 vs. ≤ 0.1, β: 0.931, 95%CI: 0.881 to 0.980), HVA (>0.2 vs. ≤ 0.2, β: 0.465, 95%CI: 0.404 to 0.526), CA (β: 0.155, 95%CI: 0.109 to 0.200), age (β: 0.011, 95%CI: 0.006 to 0.017), stereoacuity (>100 vs. ≤ 100, β: 0.088, 95%CI: 0.056 to 0.121), indoor near work time per day on weekends (≥8 vs. <4, β: 0.056, 95%CI: 0.004 to 0.107) may contribute to a larger difference in inter-eye SE. Additionally, outdoor activity time per day on weekdays (≥1 vs. <1, β: −0.040, 95%CI: −0.072 to −0.008) negatively impacts on it.

**Table 4:** Multivariate analysis of generalized linear models to estimate the relationship between the difference in inter-eye SE and associated factors among all children (*n* = 4,198).

**Associated Factors**	**Difference in inter-eye SE (D)**	**Univariate analysis**			**Multivariate analysis**		
		β	**95%CI**	***P*** **-value**	β	**95%CI**	***P*-value**
**Difference in inter-eye AL (mm)**							
							
≤ 0.1	0.21 ± 0.19	0^a^			0^a^		
0.1–0.3	0.32 ± 0.30	1.119	1.081 to 1.158	0.000	0.073	0.004 to 0.106	0.000
>0.3	1.35 ± 1.06	3.128	2.988 to 3.276	0.000	0.931	0.881 to 0.980	0.000
**Difference in inter-eye HVA (LogMAR)**							
≤0.2	0.35 ± 0.46	0^a^			0^a^		
>0.2	1.28 ± 1.24	2.585	2.407 to 2.776	0.000	0.465	0.404 to 0.526	0.000
**Age (years)**	0.42 ± 0.61	1.062	1.056 to 1.069	0.000	0.012	0.005 to 0.019	0.001
**Difference in inter-eye CA (D)**	0.42 ± 0.61	1.439	1.355 to 1.529	0.000	0.155	0.109 to 0.200	0.000
**Stereoacuity (arc-s)**							
≤100	0.36 ± 0.47	0^a^			0^a^		
>100	0.56 ± 0.83	1.217	1.165 to 1.271	0.000	0.088	0.056 to 0.121	0.000
**Indoor near work time per day on weekends (hours)**							
<4	0.35 ± 0.51	0^a^			0^a^		
4–8	0.43 ± 0.60	1.094	1.046 to 1.143	0.000	−0.006	−0.040 to 0.028	0.733
≥8	0.63 ± 0.83	1.337	1.261 to 1.417	0.000	0.056	0.004 to 0.107	0.035
**Outdoor activity time per day on weekdays (hours)**							
<1	0.48 ± 0.70	0^a^			0^a^		
≥1	0.37 ± 0.53	0.893	0.857 to 0.930	0.000	−0.040	−0.072 to −0.008	0.014

Multivariable from generalized linear models were adjusted for gender, refractive status of the worse eye, indoor near work time per day on weekdays (hours) and outdoor activity time per day on weekends (hours). SE, Spherical equivalent; AL, Axial length; HVA, Habitual visual acuity; CA, Corneal astigmatism; D, Diopter; mm, Millimeter; CI, Confidence interval; 0^a^, Reference group.

**Figure 3:**
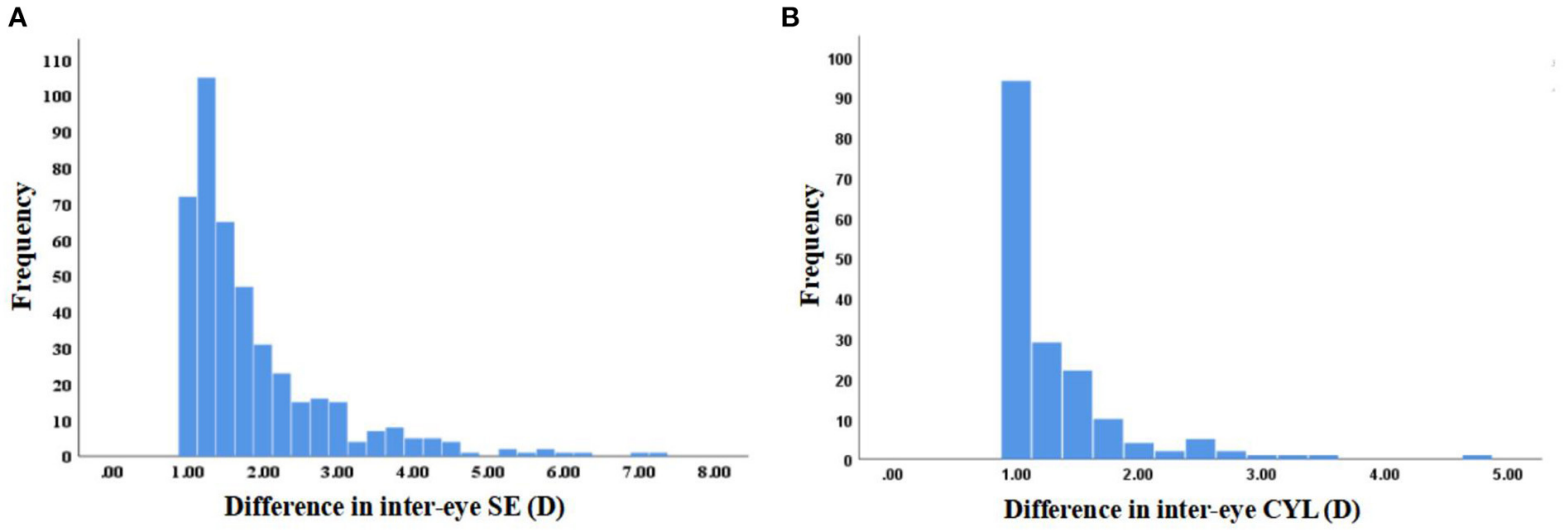
Distribution of difference in inter-eye spherical equivalent (SE) and cylinder (CYL) in the SE **(A)** and CYL **(B)** anisometropia group.

In the SE and CYL anisometropia group, the mean difference in inter-eye SE and CYL were 1.82 ± 1.01 D (median: 1.44 D; range: 1.00–7.25 D) and 1.31 ± 0.54 D (median: 1.00 D; range: 1.00–4.75 D). The distribution is shown in Figure 3. In the SE anisometropia group, using multivariate analysis, the difference in inter-eye AL (>0.3 vs. ≤ 0.1, β: 0.556, 95%CI: 0.050 to 1.063), HVA (>0.2 vs. ≤ 0.2, β: 0.511, 95%CI: 0.312 to 0.710), and CA (β: 0.488, 95%CI: 0.289 to 0.688), stereoacuity (>100 vs. ≤ 100, β: 0.299, 95%CI: 0.110 to 0.488) had a positive impact on the outcome (shown in Table 5). Mean difference in inter-eye SE was 1.67 ± 0.85 D (median: 1.38 D; range: 1.00–7.25 D) and 1.86 ± 1.09 D (median: 1.38 D; range: 1.00–5.88 D) with a prevalence of 6.8% (286/4198, 95% CI: 6.1 to 7.6%) and 2.3% (98/4198, 95%CI: 1.9 to 2.8%) in myopic and hyperopic anisometropia group. Compared to hyperopic anisometropia (18.4%, 98/553), myopic anisometropia was the most prevalent (51.7%, 286/553).

**Table 5:** Multivariate analysis of generalized linear models to estimate the relationship between the difference in inter-eye SE and ocular or lifestyle parameters among in the SE anisometropia group (*n* = 432).

**Associated Factors**	**Difference in inter-eye SE (D)**	**Univariate analysis**			**Multivariate analysis**		
		β	**95%CI**	***P*-value**	β	**95%CI**	***P*-value**
**Difference in inter-eye AL (mm)**							
≤0.1	1.19 ± 0.38	0^a^			0^a^		
0.1–0.3	1.23 ± 0.65	0.013	−0.611 to 0.636	0.968	0.034	−0.538 to 0.606	0.907
>0.3	1.92 ± 1.03	0.689	0.138 to 1.240	0.014	0.556	0.050 to 1.063	0.031
**Difference in inter-eye HVA (LogMAR)**							
≤0.2	1.61 ± 0.83	0^a^			0^a^		
>0.2	2.21 ± 1.19	0.623	0.414 to 0.833	0.000	0.511	0.312 to 0.710	0.000
**Difference in inter-eye CA (D)**	1.82 ± 1.01	0.551	0.335 to 0.766	0.000	0.488	0.289 to 0.688	0.000
**Stereoacuity (arc-s)**							
≤100	1.59 ± 0.82	0^a^			0^a^		
>100	2.05 ± 1.13	0.428	0.224 to 0.632	0.000	0.299	0.110 to 0.488	0.002

Multivariable from generalized linear models were adjusted for the outdoor activity time per day on weekends (hours). AL, Axial length; HVA, Habitual visual acuity; CA, Corneal astigmatism; mm, Millimeter; D, Diopter; CI, Confidence interval; 0^a^, Reference group.

## Discussion

Anisometropia is a type of refractive error and is a common cause of amblyopia that affects binocular vision. However, the etiology and pathogenesis have not been fully clarified. Improving the detection rate of anisometropia and early intervention and treatment are still the key points to reducing its harm. We investigated the prevalence of anisometropia and associated factors in school-aged children. The results showed that ocular parameters and lifestyle parameters were associated to anisometropia. Ocular parameters such as the refractive state of the worse eyes, the difference in inter-eye AL, HVA, CA, and lifestyle parameters such as indoor near work time per day on weekends and outdoor activity time per day on weekdays were associated with the occurrence of anisometropia.

The prevalence of anisometropia (difference in inter-eye SE or CYL ≥ 1.00 D) was 13.2%, and myopic anisometropia was the most prevalent (51.7%), which was in good agreement with research results in China (4). Since there was no national multi-center large sample study in China, the high prevalence of anisometropia might be related to the high prevalence of myopia, as well as the race, age, and different diagnostic criteria of anisometropia (1). The results showed that the prevalence and severity of anisometropia increased with age. We think it may be caused by the increased burden on children's eyes (8, 9).

### Associated factors of anisometropia

We found that anisometropia was more common in hyperopic and myopic children, and more myopic and hyperopic refractive errors in one eye were associated with the occurrence of anisometropia, which has also been validated in other studies (10, 11). The greater difference in the speed of emmetropia and myopia, the greater the possibility of anisometropia, which may be due to the failure of the regulation of the inter-eye homeostatic mechanism (12). It was suggested that preventing myopia and early treating anisometropic amblyopia could be an effective way to reduce the prevalence of anisometropia.

The difference in inter-eye AL was associated with the occurrence of anisometropia in our study, which confirmed that the unbalanced development of two eyes was the main reason for anisometropia in the previous study (13). A cross-sectional study (13) found that differences in the growth rate of the AL were the main factors in the development of anisometropia, which validated our conclusion. Astigmatism in children is mainly from corneal astigmatism (CA). The difference in inter-eye CA may be attributed to increased AL and changes in posterior scleral structure affecting the cornea at the limbus (14). The difference in inter-eye CA would lead to the occurrence of anisometropia and the further development of the difference in inter-eye SE. The results showed that the larger difference in inter-eye CA was associated with anisometropia in our study. In other words, if children with differences in inter-eye AL and CA were found, special attention should be paid to the possibility of anisometropia in clinical diagnosis.

In our study, after adjusting the refractive error and difference in inter-eye AL, the difference in inter-eye HVA was strongly associated with anisometropia. HVA was more conducive to visual performance in everyday conditions (either with or without optical correction) (15). The difference in inter-eye HVA≥0.2 logMAR indicated a higher risk of anisometropia. Previous studies also have shown that the severity of anisometropia was positively correlated with visual impairment (16, 17). Retinal image blur and unequal images caused by anisometropia were the main reasons affecting stereoscopic vision (17). Therefore, when these symptoms are found, it is recommended that ophthalmologists and optometrists take measures to intervene in advances, such as eliminating the form deprivation and abnormal interaction between the eyes (eliminating the inhibition of non-amblyopic eyes on amblyopic eyes).

### Early intervention in the lifestyle of anisometropia

Goldschmitt (18) speculated that environmental factors might affect the symmetry of binocular refraction. Some etiological studies (9, 19) also found that long time spent in near work was a risk factor for anisometropia. In our study, after adjusting for factors such as age and AL, the larger anisometropia was associated with indoor near work time per day on weekends and outdoor activity time per day on weekdays, which was consistent with our previous findings (4).

When children engaged in near work, such as daily reading and writing, the eyes with more myopic refraction were better to work at a near distance [20]. The difference between inter-eye accommodation demands may provide stimulation for the asymmetric growth of the eyes, which could accelerate the process of myopia in the worse eye, resulting in a larger difference in inter-eye SE and the occurrence of anisometropia. For example, when the working distance was close and the head was tilted, it was assumed that the eyes with lower binocular accommodation requirements had a consistent adaptive response. It might lead to hyperopic defocus in eyes with lower accommodation demand (9). The state of the object image focused on the retina was different. The differences can result in different image quality, which could provide stimulation for the occurrence of anisometropia. Thus, indoor near work time and outdoor activity time may be associated with the occurrence of anisometropia. Children's lifestyles are difficult to accurately record, and the evidence related to lifestyle research is not yet clear. Our study is not strong enough to address this issue. The association can be studied in future research.

Strengths of this study included a school-based design which had a relatively large sample size and the availability of multivariate confounding factors adjusted in the model. The subgroup analyses further supported the robustness of the study findings. HVA more accurately reflects children's daily visual state and was included in the discussion of associated factors. In our study, SE and CYL anisometropia were considered, which the latter being disregarded in several studies. Several limitations should also be noted. Firstly, constrained by cross-sectional studies, no causal relationship between associated factors and anisometropia has been demonstrated. Lastly, the collection of outdoor activity time and near work time by self-reported questionnaires inevitably leads to recall bias.

In summary, we found that anisometropia in children aged 4 to 17 was likely the result of a combination of genetic and environmental factors. Preventing the occurrence of myopia and early treating anisometropic amblyopia might help to control the further development of anisometropia. Ophthalmologists and optometrists should pay attention to the changes of difference in inter-eye SE, AL, CA, and HVA, stereoacuity, to identify the current situation of anisometropia and the effect of the intervention. Indoor near work time and outdoor activity time may be associated with the occurrence of anisometropia.

## Data availability statement

The raw data supporting the conclusions of this article will be made available by the authors, without undue reservation.

## Ethics statement

The studies involving human participants were reviewed and approved by the Ethics Committee of the Affiliated Eye Hospital of Shandong University of Traditional Chinese Medicine. Written informed consent to participate in this study was provided by the participants' legal guardian/next of kin.

## Author contributions

HB and YH led the overall study, contributed to the research design, and participated in writing the manuscript. ZX, ZW, and YWe contributed to the data collection, data analysis, and manuscript edits. MD, WS, YWa, ZS, and YL collected the epidemiological and clinical data. MY and GL processed statistical data. All authors read, contributed to the research design, and approved the final manuscript.
